# Post‐curing effects on mechanical properties staining resistance of a 3D‐printed resin versus a direct composite for definitive restorations

**DOI:** 10.1111/eos.70093

**Published:** 2026-04-20

**Authors:** Bianca Rossi, Arthur Henrique Marques Massarenti, Giulia Gamero Pizzanelli, Márcia Valéria Gualberto Barbosa de Queiroz, Filipe Milazzo dos Santos, Matheus Kury, Adriano Fonseca Lima

**Affiliations:** ^1^ Dental Research Division Paulista University Sao Paulo Brazil

**Keywords:** cohesive, elastic modulus, flexural strength, polymer

## Abstract

This study evaluated the mechanical performance and staining resistance of a three‐dimensional (3D)‐printed resin (VarseoSmile Crown Plus) subjected to different post‐curing times (0, 5, 10, 15, and 20 min), compared with a conventional nanofilled resin composite (Filtek Z350XT). Specimens of both materials were fabricated and tested for flexural strength and flexural modulus using a three‐point bending test, whereas cohesive strength (CS) was assessed by microtensile testing. For color analysis, disk specimens were immersed in artificial saliva, Coca‐Cola, or red wine (RW) for 7 days, followed by polishing and whitening procedures. Color change (Δ*E*_00_) and whiteness index (ΔWI_D_) were recorded. Data were analyzed using a generalized linear model and Bonferroni tests (*α* = 5%). The direct composite showed higher flexural and CS than the 3D‐printed resin. Post‐curing improved the mechanical properties of the printed material, although values remained inferior to those of the composite. Both materials exhibited color changes after staining, with RW causing the greatest discoloration. Polishing and whitening reduced staining but did not always restore baseline values. Although post‐curing enhances 3D‐printed resin performance, its mechanical behavior and color stability remain inferior to those of conventional composites, indicating the need for further optimization before clinical use as definitive restorations.

## INTRODUCTION

Resin‐based composites are among the most widely used materials in restorative dentistry due to their versatility, esthetics, and ability to support minimally invasive approaches. Their use has expanded significantly over the past decades, providing clinicians with materials capable of restoring teeth conservatively while maintaining both function and appearance over the years [1]. Nonetheless, in cases of extensive structural loss or full‐arch rehabilitations, indirect restorations become necessary to achieve predictable outcomes in terms of strength, longevity, and esthetics [2, 3].

Computer‐aided design and computer‐aided manufacturing (CAD–CAM) technology has revolutionized the production of indirect restorations, enabling the fabrication of highly precise and reproducible restorations in a reduced timeframe. Although ceramic and resin‐based CAD–CAM blocks have been widely adopted, the subtractive nature of milling techniques inherently leads to material waste, wear of burs, and limitations in reproducing fine geometrical details [4]. In this context, additive manufacturing (AM), or three‐dimensional (3D) printing, has emerged as an alternative with the potential to overcome these limitations. Through different printing technologies, such as stereolithography, digital light processing (DLP), or liquid crystal display–based systems, 3D printing produces restorations layer by layer, enabling cost‐effective fabrication, reduced waste, and greater design freedom [4, 5, 6].

Initially, printable resins were designed for temporary or interim applications [7, 8, 9]. More recently, however, materials specifically indicated for definitive restorations have become commercially available [10, 11, 12]. These resins, often containing higher filler loads and optimized photoinitiator systems, are expected to provide enhanced mechanical resistance and improved stability compared with earlier generations [10]. Nevertheless, the clinical suitability of such materials remains under debate. Recent investigations have shown that, although 3D‐printed resins may achieve acceptable mechanical properties, their long‐term clinical behavior is less predictable, with concerns regarding fracture resistance, wear, and discoloration [13, 14]. Indeed, clinical studies have reported that even reinforced 3D‐printed resins may undergo significant wear or cohesive fractures over relatively short observation periods, suggesting that some of these materials may still be more suitable as long‐term provisional rather than definitive solutions [14].

For a material to be clinically suitable for permanent restorations, it must withstand masticatory forces without undergoing deformation or fracture, maintain its mechanical performance over time, and resist staining from dietary pigments. Color stability is a particularly relevant property, as staining from beverages, such as wine and soft drinks, may compromise esthetics and patient satisfaction [12, 15]. If discoloration occurs, it should be reversible through routine clinical procedures, such as polishing or bleaching. To date, only few studies have evaluated the impact of repolishing and/or tooth whitening on stained 3D‐printed resin [16, 17, 18]. Furthermore, factors inherent to the AM workflow—including layer thickness, printing orientation, post‐processing, and post‐curing time—can substantially influence the mechanical and optical properties of 3D‐printed resins [11, 12]. Studies have demonstrated that inadequate post‐curing may compromise both the degree of conversion (DC) and the dimensional stability of restorations, potentially leading to inferior performance [4, 7, 8, 9, 11, 19]. However, most of the available evidence has focused on temporary resins or definitive resins with relatively low filler content.

Despite the increasing clinical adoption of 3D‐printed restorative materials, there is a scarcity of studies assessing the performance of high‐filler, definitive resins under conditions relevant to long‐term clinical use. In particular, data regarding their mechanical properties and color stability compared with well‐established direct restorative composites remain limited. Therefore, the aim of this study was to evaluate the flexural strength (FS), elastic modulus, cohesive strength (CS), and staining resistance of a commercially available 3D‐printed resin indicated for definitive restorations. Moreover, this research sought to evaluate the impact of repolishing and in‐office bleaching on color of the 3D‐printed resin. A widely used nanoparticulate resin composite, extensively validated in clinical practice, was selected as the control material. The null hypothesis (H_0_) tested were that the 3D‐printed resin would exhibit comparable [1] mechanical performance and [2] color stability to the conventional composite.

## MATERIAL AND METHODS

### Experimental design

This in vitro study comprised two main evaluations: (1) mechanical properties (*n* = 12/group) and (2) colorimetric analysis (*n* = 10/group). First, the mechanical properties (FS, elastic modulus, and CS) of a 3D‐printed resin were analyzed after different post‐curing times (0, 5, 10, 15, and 20 min). A nanoparticle resin composite, commonly used in direct restorative procedures, served as the reference material.

Subsequently, specimens of both resins, prepared under identical conditions, were randomly allocated into different 7‐day staining challenges using artificial saliva, sugar‐sweetened Coca‐Cola (CK), or red wine (RW). After immersion, the specimens underwent polishing and bleaching procedures. Color change (Δ*E*_00_) and the whiteness index for dentistry (ΔWI_D_) were assessed at different time points.

### Sample size calculation

Sample size was determined based on an a priori power analysis considering the experimental designs adopted in the present study. Assuming an alpha level of 0.05 and a statistical power of 80% and considering a moderate‐to‐large effect size based on previous studies evaluating similar properties in resin‐based materials, the analysis indicated that approximately 8–10 specimens per experimental condition would be sufficient to detect statistically significant differences. Therefore, 10 or 12 specimens per group were included depending on the experimental test.

### Specimen preparation

Three types of specimens were prepared: bar‐shaped (for flexural testing), hourglass‐shaped (for CS), and disk‐shaped (for colorimetric analysis). Designs were created with open‐source CAD software (Meshmixer, Autodesk) and exported as standard tessellation language files. These files were imported into the printer software, where support structures were added.

For all tests, specimens were printed in a horizontal orientation (0° build angle) using a DLP printer (Photon Mono X, Anycubic) at a layer thickness of 50 *µ*m, employing a 3D‐printed resin (VarseoSmile Crown Plus, BEGO; shade A1). After printing, specimens were removed from the build platform and ultrasonically cleaned in 96% ethanol—first for 3 min (coarse cleaning) and then for 2 min—followed by ethanol spray and oil‐free air drying, according to the manufacturer's instructions. Post‐curing was performed immediately using a Wash & Cure 3.0 Plus unit (Anycubic) for the designated times (0, 5, 10, 15, and 20 min).

Reference material specimens (Filtek Z350XT, Solventum; shade A1) were inserted into stainless steel molds, covered with a thin glass slide (∼0.1 mm) to ensure a flat surface and minimize the oxygen‐inhibited layer. Polymerization was performed with a dual‐peak LED curing unit (Bluephase 2G, Ivoclar) at 1200 mW/cm^2^ for 20 s per exposure. The compositions of the materials used are provided in Table 1.

**TABLE 1:** Composition of resin materials used in the study according to the Material Safety Data Sheet provided by the manufacturers.

Material	Composition
**VarseoSmile Crown Plus** (BEEGO, Bremen, Germany)	Esterification products of 4.4′‐isopropylidiphenol, ethoxylated and 2‐methylprop‐2enoic acid. Silanized dental glass, methyl benzoylformate, diphenyl (2,4,6‐trimethylbenzoyl) phosphine oxide Total content of inorganic fillers (particle size 0.7 *µ*m) is 30%–50% by mass
**Z350XT** (Solventum, St. Louis, MI, USA)	Organic phase: UDMA, Bis‐GMA, Bis‐EMAans TEGDMA Inorganic matrix: Silica (20 nm nonagglomerated/aggregated and agglomerated), clusters, zirconia/silica aggregated particles (78.5 wt%, 20 nm silica particles combined with 4–11 nm zirconia 3)

### Flexural strength (FS) and modulus (*E*)

Flexural properties were evaluated using a three‐point bending test (*n* = 12). Bar‐shaped specimens (25 mm × 2 mm × 2 mm) were obtained either from 3D‐printing or from stainless steel molds (Odeme Dental Research) for the reference material. The resin composite was inserted into the mold on a glass plate and covered with a 0.1‐mm glass slide. Polymerization was performed with the curing light tip placed centrally and flat against the specimen axis, using five overlapping irradiations (5 × 20 s).

Specimen dimensions were measured before testing with a digital caliper (Mitutoyo; ±0.01 mm). The bending test was performed in a universal testing machine (2000RK, Kratos) at 1 mm/min crosshead speed, with a support span of 20 mm.

FS was calculated as

$$\text{FS}=3FI/\left(2bh"2"\right)$$

where *F* is the maximum load at fracture (N), I is the support span (20 mm), *b* is the specimen width, and *h* is the specimen thickness.

Flexural modulus (*E*) was calculated from the slope of the stress–strain curve using

$$E=\Delta F/\Delta Y\times I``3^{\prime \prime }/\left(4bh``3^{\prime \prime }\right)$$

where Δ*F*/Δ*Y* is the load change per unit deflection at the specimen center, I is the support span, *b* is the width, and *h* is the thickness.

### Cohesive strength (CS)

For the preparation of direct resin composite specimens, hourglass‐shaped specimens (7 mm height, 3 mm width, with a 1 mm × 1 mm constriction) were prepared using stainless steel molds (Odeme Dental Research) (*n* = 12). After 24 h storage at 37°C, specimens were tested in a microtensile device (Odeme Dental Research) attached to a universal testing machine at 0.5 mm/min. The fractured area was measured with a digital caliper (Mitutoyo).

CS (MPa) was calculated as

$$\mathrm{CS}=\frac{F\times 9.8}{\mathrm{Area}}$$

### Staining challenges

Disk‐shaped specimens (5 mm in diameter × 1 mm in thickness) were subjected to staining after post‐curing and compared with the reference composite. All specimens, including those of the reference material, were polished using abrasive spiral tips (medium and fine grit, Jiffy Original Composite Polishing System, Ultradent) at low rotation. Baseline color measurements were obtained using a spectrophotometer (VITA Easyshade V, VITA Zahnfabrik).

Specimens were individually immersed in 10 mL of one of the following solutions:

*Artificial saliva (AS)*: prepared fresh according to Mota *et al*. [20] (1.5 mM CaCl_2_, 0.9 mM Na_3_PO_4_, and 0.15 mM KCl; pH 7.0).
*CK*: sugar‐sweetened (Atlanta, GA, USA).
*RW*: Tinto Demisec (Marques de La Colina, Mendoza, Argentina).

Solutions were renewed after 4 days, following Silva *et al*. [21]. After 7 days, specimens were rinsed with distilled water, dried, and reevaluated for color.

### Polishing and bleaching procedures

Following staining, specimens were repolished with the Jiffy system (medium‐ then fine‐grit, 20 s each) using low rotation. A new color measurement was obtained.

Next, the same polished surface was subjected to two sessions of in‐office bleaching with 35% hydrogen peroxide gel (Whiteness HP, FGM). The gel was prepared per manufacturer instructions (three drops peroxide solution:one drop thickener), homogenized, and applied as a 1 mm layer for 45 min without renewal [22]. After each session, specimens were stored in artificial saliva at controlled humidity. A final color evaluation was performed after the second session.

### Colorimetric evaluation

Color measurements were performed with the VITA Easyshade V spectrophotometer, based on the CIE Lab* system. Readings were performed in four stages: baseline—after the post‐cure or 24 h from the light‐curing of the resins (**T_0_**), after staining challenges (**T_1_**), after polishing procedures (**T_2_**), and after the second in‐office bleaching session (**T_3_**). The color change was calculated using the Δ*E*_00_ and ΔWI_D_ parameters. The color variation was evaluated using the CIEDE2000 formula (Δ*E*_00_):

$$\Delta E_{00}=\sqrt{{\left(\frac{\Delta L^{\prime }}{K_{L}SL}\right)}^{2}+{\left(\frac{\Delta C^{\prime }}{K_{C}SC}\right)}^{2}+{\left(\frac{\Delta H^{\prime }}{K_{H}SH}\right)}^{2}+RT\left(\frac{\Delta C^{\prime }}{K_{C}SC}\right)\left(\frac{\Delta H^{\prime }}{K_{H}SH}\right)}$$

The variation in the WI_D_ was calculated using the following formula:

$$WI_{D}=\left[\left(0.511\times L\right)-\left(2.324\times a*\right)-\left(1.100\times b*\right)\right]$$

where Δ*E*_00_ and ΔWI_D_ were calculated taking into account different time point intervals. The Δ*E*_00_ values were assessed using the perception threshold (PT) and the acceptance threshold (AT), with values of 0.81 and 1.8 units, respectively. Similarly, the ΔWI_D_ changes were compared against PT and AT thresholds of 0.7 and 2.6, respectively [23].

### Statistical analyses

Data were analyzed using spss software (version 23; IBM). For mechanical and CS data, each experimental condition was treated as an independent group, as the post‐curing protocol was applied only to the 3D‐printed resin and not to the conventional composite. Therefore, comparisons among groups were performed using a generalized linear model considering a single factor (“resin/processing condition”), followed by Bonferroni post hoc tests (*α* = 0.05).

Colorimetric data were analyzed using a generalized linear model including two factors (“resin” and “staining condition”) to evaluate their main effects and interaction. Pairwise comparisons were performed using the Bonferroni test (*α* = 0.05).

## RESULTS

### Mechanical properties

**FIGURE 1:**
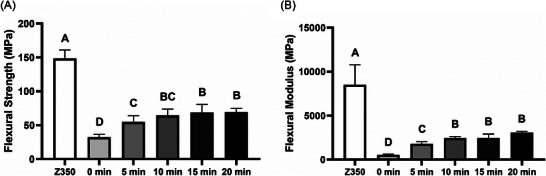
Mean and standard deviation of (A) flexural strength and (B) flexural modulus. Distinct uppercase letters indicate statistical differences, taking into consideration generalized linear models followed by Bonferroni post hoc testing (*α* = 5%).

Figure 1 presents the mean and standard deviation values for FS (A) and flexural modulus (*E*) (B). The factor *resin* significantly affected both outcomes (*p* < 0.001), with the composite resin (Z350) showing the highest FS and modulus. Among the 3D‐printed resins, post‐curing for 15 or 20 min yielded intermediate values. Although 10 min of post‐curing resulted in FS values comparable to those of 15 and 20 min, it was also statistically similar (*p* > 0.05) to 5 min, which in turn was significantly lower than 15 and 20 min. For flexural modulus, no significant differences were observed among the 10‐, 15‐, and 20‐min post‐curing groups, all of which were significantly higher than the 5‐min group. In both outcomes, the absence of post‐curing (0 min) produced the lowest FS values.

**FIGURE 2:**
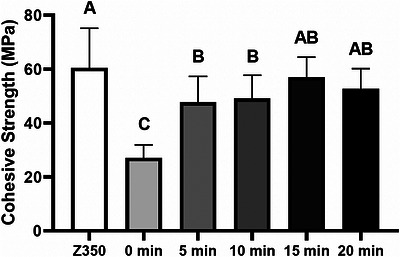
Mean and standard deviation of cohesive strength. Distinct uppercase letters indicate statistical differences, taking into consideration generalized linear models followed by Bonferroni post hoc testing (*α* = 5%).

Figure 2 presents the mean and standard deviation values of CS. Statistical analysis revealed a significant effect of the factor *resin* (*p* < 0.001). Pairwise comparisons indicated that the composite resin (Z350) achieved the highest mean values, with no significant differences compared to 3D‐printed resins post‐cured for 15 and 20 min. Post‐curing for 5 and 10 min yielded values statistically similar to those obtained at 15 and 20 min (*p* > 0.05), but significantly higher than the non–post‐cured 3D resin group, which exhibited the lowest CS (*p* < 0.05).

### Color and whiteness changes

#### Baseline coordinates

**FIGURE 3:**
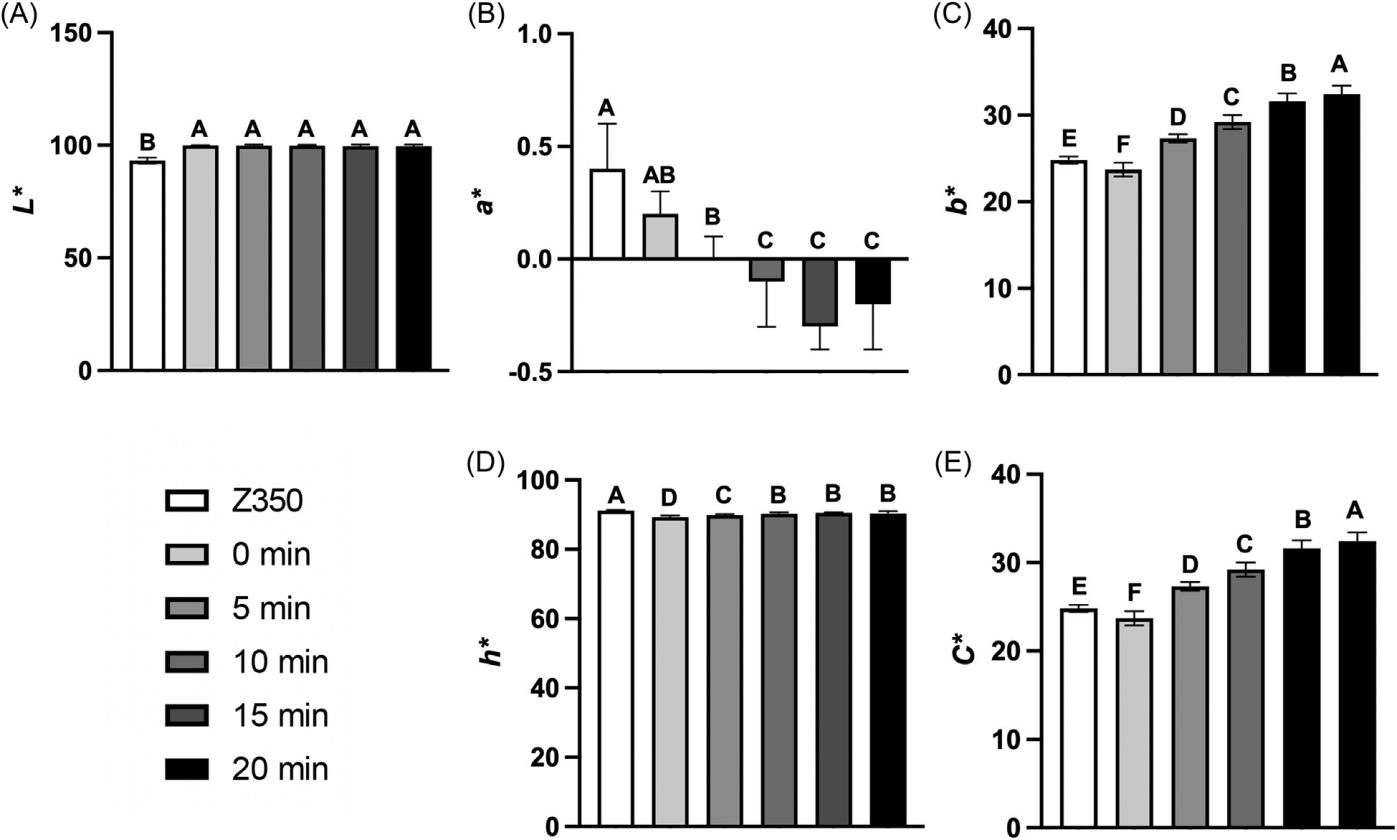
Mean and standard deviation of coordinates after different post‐curing times: (A) lightness (L*), (B) red‐green axis (a*), (C) yellow‐blue axis (b*), (D) hue (h*), and (E) chroma (C*). Uppercase letters compare post‐curing times within each parameter. Generalized linear models with Bonferroni (*α* = 5%).

Figure 3 shows the individual parameters (L*, a*, b*, h*, and C*) of Z350 and 3D‐printed resins subjected to different post‐curing times. Lightness (L*) remained stable regardless of post‐curing time, with no significant differences among printed groups, whereas Z350 presented slightly lower L* values (*p* < 0.05). The a* coordinate was significantly higher for Z350, and a progressive reduction in redness was observed in 3D‐printed resins as post‐curing time increased, with the lowest a* values recorded after 10, 15, and 20 min. The b* coordinate increased significantly with longer post‐curing, indicating a more pronounced yellow component, with the highest values after 20 min (*p* < 0.05). Similarly, chroma (C*) increased progressively as post‐curing time increased, suggesting higher color saturation in most cured materials. Hue (h*) decreased after post‐curing compared with Z350, stabilizing after 15 min. Overall, these results indicate that extended post‐curing promotes a shift toward more saturated and yellowish shades with reduced red components, whereas lightness remains unaffected.

#### After staining versus baseline

**FIGURE 4:**
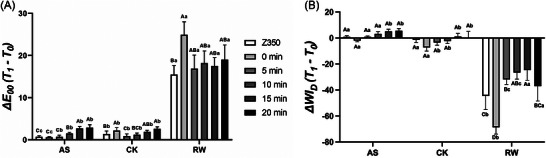
Mean and standard deviation of Δ*E*_00_ (A) and ΔWI_D_ (B) between baseline (T_0_) and after staining (T_1_) time points. Uppercase letters compare the different resins within the same staining level. Lowercase letters compare the same resin among the different staining levels. Generalized Linear Models with Bonferroni (*α* = 5%).

**FIGURE 5:**
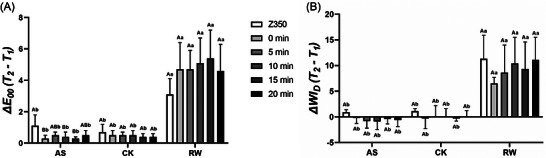
Mean and standard deviation of Δ*E*_00_ (A) and ΔWI_D_ (B) between after staining (T_1_) and after polishing (T_2_) time points. Uppercase letters compare the different resins within the same staining level. Lowercase letters compare the same resin among the different staining levels. Generalized Linear Models with Bonferroni (*α* = 5%).

**FIGURE 6:**
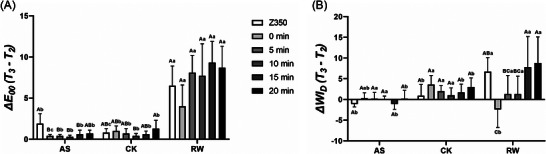
Mean and standard deviation of Δ*E*_00_ (A) and ΔWI_D_ (B) between after polishing (T_2_) and after bleaching (T_3_) time points. Uppercase letters compare the different resins within the same staining level. Lowercase letters compare the same resin among the different staining levels. Generalized Linear Models with Bonferroni (*α* = 5%).

Both factors, *resin* and *staining*, significantly influenced the results, with an interaction between them (*p* < 0.05) across all time point comparisons demonstrated in Figures 3, 4, 5, 6. Figure 4 illustrates that regardless of the resin, RW staining produced significantly higher Δ*E*_00_ and ΔWI_D_ compared to AS and CK. Under RW, the 3D‐printed without post‐curing presented significantly higher Δ*E*_00_ than Z350. Although no significant differences were detected among all the 3D‐printed groups, those submitted to post‐cure (5–20 min) were also similar to Z350 and presented differences to 0 min that were higher than the perceptibility and acceptability threshold.

All RW‐stained resins yielded negative ΔWI_D_ values, with the non–post‐cured 3D resin (0 min) showing the greatest reduction in whiteness (*p* < 0.05). The smallest reduction was observed in 3D resins post‐cured for 10 and 15 min (*p* < 0.05), whereas the 5‐ and 20‐min groups, as well as Z350, demonstrated intermediate values, all significantly different from the 0 min group. Under AS staining, 10‐, 15‐, and 20‐min resins achieved significantly higher Δ*E*_00_ than the other resins, but corresponding mean ΔWI_D_ showed no significant differences among all resins. For CK, only Δ*E*_00_ presented significant differences, on the contrary of ΔWI_D_, whose mean values were negative in most groups.

All resins immersed in RW and the 0‐min post‐cured 3D resin under CK presented both mean Δ*E*_00_ and ΔWI_D_ (negative) above the PT and AT. In AS‐stained resins, only 15‐ and 20‐min post‐cured 3D resins exhibited mean Δ*E*_00_ and ΔWI_D_ above the AT, but mean ΔWI_D_ was positive in this occasion.

#### After polishing versus after staining

Figure 5 shows that, after polishing, Δ*E*_00_ and ΔWI_D_ were significantly higher in RW‐stained resins, irrespective of post‐cure time or resin type. No significant differences in Δ*E*_00_ and ΔWI_D_ were observed among resins within each staining condition, except for AS‐stained samples, where a difference was detected between Z350 and 0 min, but only for Δ*E*_00_ (*p* < 0.05). In 3D‐printed resins, polishing resulted in positive mean ΔWI_D_ only under RW staining. Z350 exhibited positive mean ΔWI_D_ regardless of staining; however, RW staining produced not only significantly higher ΔWI_D_ values but also values far exceeding both perceptibility and acceptability thresholds.

#### After bleaching versus after polishing

Figure 6 shows that bleaching resulted in the highest Δ*E*_00_ values (*p* < 0.05) in RW‐stained resins, with no significant differences among the resins within this staining condition. Notably, the 0‐min 3D‐printed resin stained with RW exhibited lower mean Δ*E*_00_, whose differences to all other resins exceeded both perceptibility and acceptability thresholds. This group also presented negative ΔWI_D_ values, which were comparable only to the 3D‐printed resins post‐cured for 5 and 10 min (*p* > 0.05). Resins post‐cured for 15 and 20 min showed significantly higher mean positive ΔWI_D_ values compared with 0 min and were similar to Z350, whose ΔWI_D_ was also comparable to the 5‐ and 10‐min post‐cure groups. Although significant Δ*E*_00_ differences were detected among resins in the AS‐ and CK‐stained groups, bleaching‐induced ΔWI_D_ values were similar across all resins (*p* > 0.05).

#### After bleaching versus baseline

**FIGURE 7:**
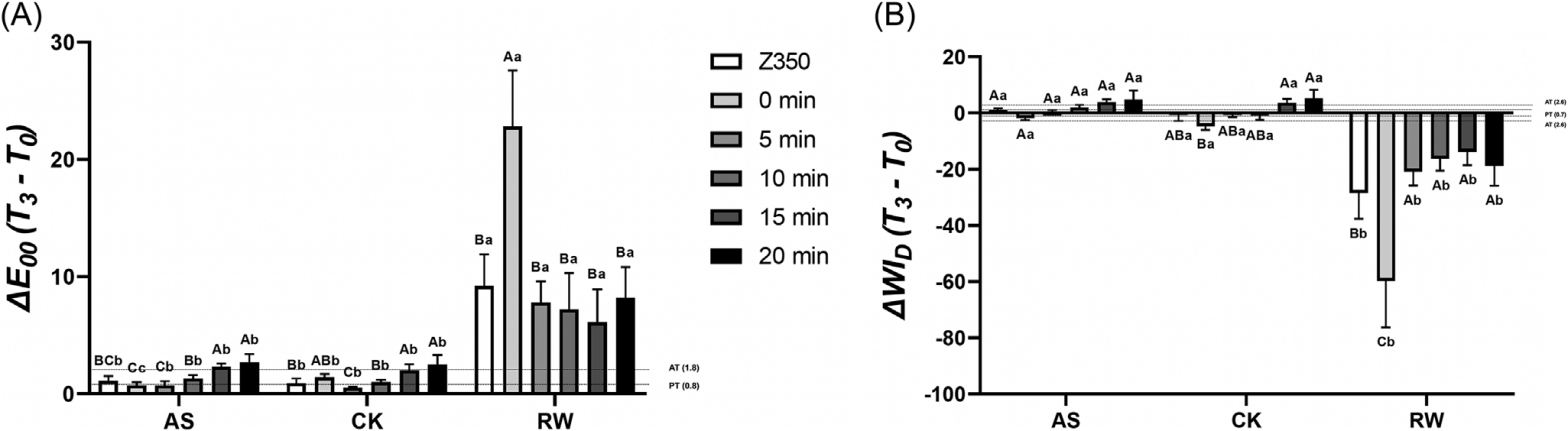
Mean and standard deviation of Δ*E*_00_ (A) and ΔWI_D_ (B) between baseline (T_0_) and after bleaching (T_3_) time points. Uppercase letters compare the different resins within the same staining level. Lowercase letters compare the same resin among the different staining levels. Generalized Linear Models with Bonferroni (*α* = 5%).

Figure 7 shows that, when comparing the first and last time points, Δ*E*_00_ was significantly higher in RW‐stained resins compared with AS and CK, irrespective of resin type or post‐cure time (*p* < 0.05). In RW, the 0‐min 3D‐printed resin exhibited the highest Δ*E*_00_. In contrast, no consistent differences were observed among resins within AS and CK staining conditions, with only slight variations detected.

For ΔWI_D_, bleaching after RW still resulted in negative values for all resins, with the 0‐min 3D‐printed resin showing the greatest decrease, significantly different from all other groups (*p* < 0.05). Post‐cured resins also exhibited negative ΔWI_D_ values, but Z350 led to significantly higher decrease in whiteness. Under AS and CK staining, bleaching produced similar ΔWI_D_ values across all resins, with minimal differences among post‐cure times.

After staining, polishing, and bleaching, all RW‐stained resins presented Δ*E*_00_ and negative ΔWI_D_ that were above the AT. Interestingly, 15‐ and 20‐min post‐cured resins stained with AS and CK presented Δ*E*_00_ and positive ΔWI_D_ that were above the PT and AT. Under staining with saliva and cola‐based beverage, only Z350 and 5‐ and 10‐min post‐cured 3D resins presented mean Δ*E*_00_ and ΔWI_D_ below the AT.

## DISCUSSION

This study compared a 3D‐printed resin (VarseoSmile Crown Plus) with a well‐established direct composite (Z350XT) under different post‐curing regimens to assess their potential for definitive restorations. Because Z350 holds a long clinical history, it served as the benchmark against which the 3D resin was evaluated. The tested properties (FS, modulus, CS, and color stability) are key factors for long‐term intraoral use.

Mechanical integrity is essential for restorative materials to withstand functional loading and prevent failure. FS and *E* reflect bulk resistance to bending, whereas CS probes the integrity of the resin matrix/filler network. FS and *E* followed the same trend: Z350 achieved the highest values; the 3D resin post‐cured for 15–20 min performed at an intermediate but comparable level; 10 min showed transitional results; 5 min was weaker; and no post‐cure (0 min) produced the lowest outcomes. Therefore, the first null hypothesis was rejected.

These patterns possibly are possibly linked to the DC. Without post‐curing, the network remains underdeveloped, with many unreacted methacrylate groups, resulting in poor stiffness and cohesion [24, 25, 26]. Incremental post‐curing enhances polymerization, reduces residual monomer, improving strength [11]. The plateau observed between 10 and 20 min suggests that most conversion occurs within the first 10 min, with only marginal reinforcement afterward. Importantly, after 15–20 min, the 3D resin reached CS values comparable to Z350. The positive influence of post‐curing is consistent with recent systematic review [27] and in vitro studies showing the decisive role of post‐curing on printed resin performance [11, 28].

Direct composites are cured in bulk under high‐intensity light, in contrast with 3D‐printed resins, which is polymerized layer by layer under less homogeneous exposure, which may promote lower crosslink density compared to increased post‐cure times [29]. Although speculative, this may explain part of the mechanical gap observed and should be investigated in future studies.

Composition further influences performance. Z350 contains high‐molecular‐weight monomers (UDMA, Bis‐GMA, Bis‐EMA), known to enhance rigidity and network strength [30, 31, 32]. To remain printable, VarseoSmile omits or limits these monomers, sacrificing some mechanical reinforcement. Even more relevant is filler content. VarseoSmile contains 30–50 wt% inorganic fillers (∼0.7 µm) (BEGO), whereas Z350 reaches ∼72.5 wt% (55.6 vol%) in translucent shades and up to ∼78.5 wt% (63.3 vol%) in others (Solventum Z350 XT Brochure). This volumetric difference is critical: Higher filler volume distributes stress more effectively, stiffens the composite, and reduces deformation, whereas lower filler volume increases polymer content, making the resin more flexible and prone to fracture [33, 34]. Thus, the mechanical inferiority of printed groups, especially with insufficient post‐cure, likely reflects both network and compositional constraints.

Other studies on definitive 3D resins report variable results depending on test protocols, printing conditions, and resin formulations [10, 11, 28]. A recent study shows FS slightly higher than obtained in the present study, with similar CS and modulus below those of conventional composites [10]. Importantly, many studies lack a benchmark control, limiting the comparison with widely used materials. By including Z350, the present results combined with previous one [12] highlight that, despite improvements with post‐curing, printed resins remain mechanically inferior to established direct composites and milled alternatives.

Color stability is equally critical, particularly for anterior restorations. In the present study, the non‐post‐cured group exhibited color and negative whiteness changes exceeding perceptibility and acceptability thresholds after staining with cola‐based and RW solutions. In this scenario, RW induced the most pronounced discoloration, although this effect was clearly mitigated by post‐curing. Likewise, Lee *et al*. [35] reported that the staining potential of RW in a 3D‐printed resin intended for interim use was progressively attenuated with increasing in post‐cure up to 20 min. Ethanol, a major component of wine, has been shown to soften the resin surface [36], increasing permeability and facilitating pigment adsorption onto the polymer matrix. On the other hand, cola‐beverage likely affected only the 0 min group due to the presence of artificial colorants, such as sulfite ammonia caramel (E150d), which have relatively larger molecular structures compared with those in RW [37].

Filtek Z350XT exhibited color and whiteness changes significantly lower and perceptible changes compared with the 3D‐printed resin without post‐curing, thereby confirming the second null hypothesis. However, post‐curing of 3D resin might assure comparable color stability to a nanofilled composite, but both situations showed pronounced darkening under wine immersion. Some previous studies have already attested that nanofilled composite presents unacceptable changes after immersion in dark beverages [38, 39]. Therefore, even a conventional composite might suffer from advanced exposure to polyphenolic and alcoholic challenges. In the present study, 7 days of staining challenges were designed to simulate the daily intake of beverages over a period of approximately 6–7 months [40]. Therefore, the stability might be significantly more compromised in a longer term evaluation.

It should be also kept in mind that prolonged post‐cure times (15 and 20 min) caused unacceptable color and whiteness changes when 3D‐printed specimens were stored in artificial saliva, but in a positive direction. In other words, the resins had a mild whitening effect even before repolishing or bleaching. A recent study pointed out that different post‐cure methods played a role in baseline color of a 3D‐printed resin indicated for long‐term indirect restorations [41]. In the present investigation, the evaluation of baseline coordinates illustrated that 15 and 20 min of post‐curing led to the highest b* and C* values, inferring higher yellowness and chroma for such groups before immersion in solutions. In other words, the 3D‐printed resin, originally displaying an A1 shade, exhibited a shift toward A3 due to increased chroma associated with longer curing period. In view of this, hydration caused by artificial saliva could have contributed for the perceptible and positive whiteness changes values.

Moreover, surface treatments proved effective: Polishing removed superficial stains, whereas bleaching degraded extrinsic pigments by breaking chromophore bonds [20]. It is important to highlight that polishing only promoted positive whiteness changes and above the PT and AT for RW‐stained resins. Souza *et al*. [18] concluded that repolishing was also effective to remove superficial staining on red‐ and coffee‐stained 3D‐resin for long‐term restorations. Similarly to our study, the authors have also observed that repolishing was not able to recover the baseline color, still leading to unacceptable color change [18]. The subsequent tooth bleaching herein performed aimed to verify whether clinicians would still have any alternative, after repolishing, to recover original color levels. Nonetheless, tooth bleaching effect was not homogenous across the groups. On top of that, the final time point comparison revealed that repolishing and bleaching together did not eliminate the unacceptable color and whiteness changes detected in all RW groups, non‐post‐cured 3D‐resin immersed in cola‐based drink, and 15‐ and 20‐min post‐cured resins stored only in artificial saliva. From the clinical standpoint, this would represent a mismatch with tooth color and the necessity of replacing of indirect restoration.

As an in vitro study, the results must be interpreted cautiously. Laboratory protocols cannot replicate the complexity of the oral environment, including fatigue, pH fluctuations, biofilm activity, and enzymatic degradation. Mechanistic factors, such as DC or network density, were inferred but not directly measured. Nevertheless, using Z350 as a reference strengthens clinical relevance compared to studies that assess printed resins in isolation.

A key question is whether the printed resin meets the ISO 4049 threshold required for definitive restorative materials. According to this standard, resin‐based materials should present a minimum FS of 80 MPa. In the present study, the 3D‐printed resin showed FS values approaching or exceeding this threshold after 15–20 min of post‐curing, whereas the conventional composite (Z350) consistently exhibited values well above this limit. In contrast, shorter post‐curing times resulted in lower mechanical performance, indicating that adequate post‐curing is essential to reach values comparable to the ISO requirement. However, it is important to recognize that meeting the minimum ISO 4049 threshold does not necessarily guarantee long‐term clinical reliability. The standard represents a minimum mechanical requirement under controlled laboratory conditions and does not account for factors such as cyclic loading, hydrolytic degradation, or long‐term fatigue behavior. For this reason, although longer post‐curing times allowed the printed resin to approach the ISO requirement, classifying these materials as definitive restorative options may still be premature.

At present, a more realistic clinical application may be as extended provisional restorations—restorations intended to remain in function longer than conventional provisionals (approximately 1–2 years), but not necessarily as permanent restorative solutions (13). As resin chemistry, filler technology, and printing protocols continue to evolve, the mechanical performance of printed resins may eventually reach levels comparable to conventional composites.

In summary, sufficient post‐curing allows the 3D resin to approach the CS and esthetic performance of Z350. However, current limitations in filler loading, network formation, and durability prevent its classification as a truly definitive material. These resins hold promise as intermediary options, whereas the technology continues to mature.

## CONCLUSIONS

Within the limitations of this in vitro study, the following conclusions can be drawn:

- The 3D‐printed resin showed mechanical properties that improved significantly with post‐curing but remained lower than those of the conventional composite. A minimum of 15 min of post‐curing was necessary to achieve values approaching the benchmark material.
- The absence of post‐curing resulted in the lowest color stability, regardless of the staining solution. Although post‐curing improved resistance to staining—reaching levels comparable to the benchmark material—RW still caused unacceptable changes in color and whiteness across all groups tested.
- Both repolishing and in‐office bleaching with hydrogen peroxide were effective in removing extrinsic stains from the resin surface. Nevertheless, these procedures did not fully restore the original color of the specimens affected by staining.
- Although the 3D resin can reach ISO threshold values under optimal post‐curing, its mechanical performance is still inferior to one well‐established direct resin composites.

## AUTHOR CONTRIBUTIONS

**Conceptualization**: Adriano Fonseca Lima, Matheus Kury, and Filipe Milazzo dos Santos. **Formal analysis**: Matheus Kury, Adriano Fonseca Lima, Márcia Valéria Gualberto Barbosa de Queiroz, Arthur Henrique Marques Massarenti, Giulia Gamero Pizzanelli, and Filipe Milazzo dos Santos. **Investigation**: Bianca Rossi, Márcia Valéria Gualberto Barbosa de Queiroz, Arthur Henrique Marques Massarenti, Giulia Gamero Pizzanelli, and Filipe Milazzo dos Santos. **Methodology**: Matheus Kury, Bianca Rossi, Márcia Valéria Gualberto Barbosa de Queiroz, Arthur Henrique Marques Massarenti, Giulia Gamero Pizzanelli, and Filipe Milazzo dos Santos. **Writing—original draft**: Bianca Rossi, Matheus Kury, and Adriano Fonseca Lima. **Writing—review and editing**: Márcia Valéria Gualberto Barbosa de Queiroz, Arthur Henrique Marques Massarenti, Giulia Gamero Pizzanelli, and Filipe Milazzo dos Santos.

## CONFLICT OF INTEREST STATEMENT

The authors declare no conflicts of interest.
